# Response of human oral mucosal epithelial cells to different storage temperatures: A structural and transcriptional study

**DOI:** 10.1371/journal.pone.0243914

**Published:** 2020-12-16

**Authors:** Mazyar Yazdani, Aboulghassem Shahdadfar, Sjur Reppe, Dipak Sapkota, Evan M. Vallenari, Majlinda Lako, Che J. Connon, Francisco C. Figueiredo, Tor Paaske Utheim

**Affiliations:** 1 Department of Medical Biochemistry, Oslo University Hospital, Ullevål, Oslo, Norway; 2 Center for Eye Research, Department of Ophthalmology, Oslo University Hospital, Ullevål, Oslo, Norway; 3 Department of Plastic and Reconstructive Surgery, Oslo University Hospital, Oslo, Norway; 4 Lovisenberg Diaconal Hospital, Unger-Vetlesen Institute, Oslo, Norway; 5 Institute of Oral Biology, Faculty of Dentistry, University of Oslo, Oslo, Norway; 6 Biosciences Institute, Faculty of Medical Sciences, Newcastle University, International Centre for Life, Bioscience West Building, Newcastle upon Tyne, United Kingdom; 7 Department of Ophthalmology, Royal Victoria Infirmary & Newcastle University, Newcastle upon Tyne, United Kingdom; 8 Department of Ophthalmology, Stavanger University Hospital, Stavanger, Norway; 9 Department of Ophthalmology, Sørlandet Hospital Arendal, Arendal, Norway; 10 Department of Computer Science, Oslo Metropolitan University, Oslo, Norway; Stein Eye Institute, University of California, Los Angeles, UNITED STATES

## Abstract

**Purpose:**

Seeking to improve the access to regenerative medicine, this study investigated the structural and transcriptional effects of storage temperature on human oral mucosal epithelial cells (OMECs).

**Methods:**

Cells were stored at four different temperatures (4°C, 12°C, 24°C and 37°C) for two weeks. Then, the morphology, cell viability and differential gene expression were examined using light and scanning electron microscopy, trypan blue exclusion test and TaqMan gene expression array cards, respectively.

**Results:**

Cells stored at 4°C had the most similar morphology to non-stored controls with the highest viability rate (58%), whereas the 37°C group was most dissimilar with no living cells. The genes involved in stress-induced growth arrest (*GADD45B*) and cell proliferation inhibition (*TGFB2*) were upregulated at 12°C and 24°C. Upregulation was also observed in multifunctional genes responsible for morphology, growth, adhesion and motility such as *EFEMP1* (12°C) and *EPHA4* (4°C–24°C). Among genes used as differentiation markers, *PPARA* and *TP53* (along with its associated gene *CDKN1A*) were downregulated in all temperature conditions, whereas *KRT1* and *KRT10* were either unchanged (4°C) or downregulated (24°C and 12°C; and 24°C, respectively), except for upregulation at 12°C for *KRT1*.

**Conclusions:**

Cells stored at 12°C and 24°C were stressed, although the expression levels of some adhesion-, growth- and apoptosis-related genes were favourable. Collectively, this study suggests that 4°C is the optimal storage temperature for maintenance of structure, viability and function of OMECs after two weeks.

## 1. Introduction

Limbal stem cell deficiency (LSCD) is a potentially painful and blinding condition caused by damage or loss of the limbal stem cells [1, 2]. Cell therapy has gained increasing attention over the last few years in the treatment of LSCD after the first successful transplantation of autologous limbal stem cells in 1997 by Italian scientists for patients with unilateral disease [3]. This therapy is believed to replace or restore remaining functioning limbal stem cells to promote regeneration of the corneal epithelium [4].

For treatment of unilateral LSCD, the use of cultured autologous limbal stem cells from the healthy cornea is often the method of choice. In the event of bilateral LSCD, which is more prevalent, the application of allogeneic limbal stem cells remains an option, but is considered less favourable because it requires systemic immunosuppressive drugs with potential adverse effects and the long-term outcome is not always satisfactory. Therefore, the quest for alternative cell types was initiated. Oral mucosal epithelial cells (OMECs) were the first alternative autologous source to be studied in both rabbits [5] and humans [6]. Later examples of cell sources include epidermal, embryonic, conjunctival epithelial, umbilical cord, hair follicle bulge, immature dental pulp, orbital fat-derived and bone marrow-derived mesenchymal stem cells [7, 8]. Of those, OMECs and conjunctival cells are hitherto the only clinically used non-limbal cell type for the treatment of LSCD [9]. Seeking to improve transplantation success and expand access to regenerative medicine, researchers have also investigated different storage conditions [10–16] and transportation techniques [17–19].

The prepared epithelial stem cells in vitro may not be used immediately for transplantation due to patient readiness and the time required for transport from laboratory units to clinics. Such a gap gives the opportunity for phenotypic investigations [20], sterility control [21] and flexible scheduling of surgery [22]. To establish optimal conditions, several studies have investigated various aspects of storage [10–16, 21, 23, 24]. These attempts are in line with the European Medicines Agency’s approval of stem cell therapy in Europe [25]. In addition, advancements in storage technology has provided the necessary practical methods for worldwide distribution of cultured cells from centralized laboratories to clinics [26] following rather strict regulations for cell therapy [27] and taking into consideration an increasing demand over the last few years [28].

Cryopreservation and refrigeration (4°C) are two common storage methods, especially for cultured epidermal cells. However, both suffer from the drawback of low cell viability [29–31]. Studies on cryopreservation have also shown fragmented cultured cell sheets with rather unsuitable morphology [23], along with complicated procedures requiring specific and costly devices. In search for an alternative and better preservation methods, our group has previously investigated the effects of different temperatures (between 4°C–37°C) on the structure and function of cultured human oral keratinocytes [16] and epidermal cells [15] for one and two week periods, respectively.

The present study aimed to examine ten significantly differentially expressed genes among the studied groups by Utheim et al. [32], and 20 genes from various relevant pathways using TaqMan gene expression array cards in cultured OMECs stored at four different temperatures (4°C, 12°C, 24°C and 37°C) for two weeks. The morphology (light and scanning electron microscopes) and cell viability (trypan blue exclusion test) of cultured epithelial cells were also investigated.

## 2. Materials and methods

### 2.1. Ethical considerations

Local ethical approval and verbal informed consent were obtained before OMEC harvesting. According to guidelines, the relevant form was completed and signed by the specialist during interview with the next of kin before transferring cadaver to the Department of Pathology, Oslo University Hospital. The research was conducted in accordance with the Declaration of Helsinki. The experimental protocols were approved by The Regional Committee for Medical and Health Research Ethics, Section C, South East Norway (reference: 2017/418).

### 2.2. Chemicals

The defined proprietary culture medium, CNT-Prime, was purchased from Cellntec Advanced Cell Systems AG (Bern, Switzerland). Dulbecco’s Modified Eagle Medium/Nutrient F-12 Ham + GlutaMAX™-I (DMEM/F12) was bought from Invitrogen Life Technologies (Carlsbad, CA, USA). Membrane inserts (Transwell cat. no. 3450) and 48-well non-tissue culture polystyrene plates (Falcon 353078) were from Corning Costar (Cambridge, MA, USA) and Becton Dickinson Labware (Franklin Lakes, NJ, USA), respectively. The rest were obtained from Sigma Aldrich (Oslo, Norway).

### 2.3. Cell isolation

Donated lower lip biopsy was harvested from cadaver’s oral cavity within 24 h post mortem in a non-refrigerated setting. The cadaver was a 56-year-old male with heart disease and good oral hygiene. The biopsy was transported to tissue-culture laboratory within 1 h in Dulbecco’s Modified Eagle Medium/Nutrient F-12 Ham + GlutaMAX™-I (DMEM/F12) supplemented with 100 U/mL penicillin-streptomycin (P/S) at room temperature and then rinsed off using the same medium. It was cut into 1 cm × 0.5 cm pieces and then incubated in Mg^2+^ and Ca^2+^-free Hanks’ balanced salt solution containing 1.2 U/mL dispase II at 37°C overnight. The separation of epithelial cell layer from the lamina propria layer was performed under a dissecting microscope by sterile forceps and scalpel. Afterwards, the tissue was rinsed with DMEM/F12, cut into explants (1–3 mm^2^), put on plastic inserts and allowed to attach in DMEM/F12 supplemented with 10% fetal bovine serum and P/S. Subsequently, the cultured cells were incubated at 37°C (5% humidified CO_2_) for two weeks. Then, the expanded cells were harvested (0.25% trypsin-EDTA solution) as passage 1 (P1) cells.

Trypan blue exclusion test was used for P1 cell counting and viability assessment before being frozen in 5% DMSO at -80°C overnight followed by liquid nitrogen storage. The OMECs were seeded on culture vessels in serum-free CNT-Prime medium supplemented with P/S on a 48-well-plate. All the cultures were incubated at 37°C and 5% CO_2_. The culture medium was changed every 2–3 days.

### 2.4. Cell storage

The frozen OMECs were thawed, seeded and expanded on culture flasks in serum-free CNT-Prime medium supplemented with P/S at 37°C with 5% CO_2_ for 5–7 days. The culture medium was changed every 2–3 days. Then, cells were trypsinized and cultured on 24-well plates with (for scanning electron microscopy) or without (for other tests) 8 mm round glass coverslips to obtain a confluent monolayer under the same condition. Before random selection for storage in cabinets at four different temperatures (4°C, 12°C, 24°C and 37°C with SD 0.4°C), the storage medium containing MEM with 12.5 mM HEPES, 3.57 mM sodium bicarbonate and 50 mg/ml gentamycin was replaced and the 24-well plates were properly sealed.

Oral mucosa epithelial cells were isolated from a single donor and cultured on uncoated plastic plates to minimize confounding variation, focus on temperature-associated differences and limit variation of scaffolds in the form of biological substrates such as amniotic membrane and fibrin [15]. Three technical replicates were used for each experiment.

### 2.5. Light microscopy

The morphology of individual cells and the integrity of the complete cell layer in all four different temperature conditions were evaluated by a Leica DMIL inverted phase contrast microscope (Leica Microsystems, Wetzlar, Germany) coupled with a Canon EOS 5D mark II camera (Canon, Oslo, Norway). The images were captured at random positions within each well at 4×, 10×, 20× and 40× magnification before (baseline) and after two weeks incubation time (4°C, 12°C, 24°C and 37°C).

### 2.6. Scanning electron microscopy

Immediately after the incubation period, fixative (PHEM 0.2M, Glutaraldehyde 2% and Paraformaldehyde 8%) pre-adjusted to desired temperature was added in a 1:1 ratio to each well. Afterwards, the plates were returned to their respective storage cabinets for 15 min. Then, fixed cells on each coverslip were dehydrated using a graded ethanol series consisting of a 10-min treatment with 70, 80, 90, and 96% ethanol, followed by 4 x 15-min treatments with 100% ethanol. Samples were then critical point dried (BAL-TEC CPD 030 Critical Point Dryer) and coverslips were mounted on SEM stubs using carbon tape. Finally, samples were sputter coated with 6 nm platinum using a Cressington 308R coating system, then all samples were examined and digital images recorded using a Hitachi S-4800 Field Emission Scanning Electron Microscope operated at 5.0 kV.

### 2.7. Cell viability test

The viability of cultured cells was evaluated by the trypan blue exclusion test [33].

### 2.8. TaqMan gene expression array cards

The cells were harvested directly in RLT buffer (Qiagen, Hilden, Germany) and mixed by pipetting up and down. The lysate was then passed through a needle (0.9-mm diameter) attached to a sterile plastic syringe 10 times for achieving a homogenous lysate. RNeasy micro kit (Qiagen, Hilden, Germany) was used for extraction and purification of total RNA. Concentration and purity of isolated RNA were assessed by spectrophotometry (Nanodrop, Wilmington, Germany). Using the High Capacity cDNA RT Kit (Applied Biosystems, Abingdon, UK), reverse transcription (RT) was performed with 200 ng of total RNA per 20 μL RT reaction based on the manufacturer’s instruction.

Comparative relative quantification was performed using the TaqMan Gene Expression Array Card for 30 genes (Table 1). The choice of genes was partly based on our previous study Utheim et al [32], in which cultured oral keratinocytes were examined for gene expression after one week storage at 4°C, 12°C and 37°C. Ten significantly differentially expressed genes (*CCNH*, *CDKN2B*, *GADD45B*, *GAS5*, *HDAC6*, *HIST2H4B*, *HUS1*, *RPSA*, *TBRG4* and *TGFB2*) among the studied groups were selected, accordingly. In addition, 19 genes from various relevant pathways were included. *GAPDH* was also included to evaluate whether energy consumption originated from oxidative phosphorylation or glycolysis.

**Table 1:** The list of genes examined using the TaqMan gene expression array in this study.

Gene Symbol	Gene Name	TaqMan Assay ID
*BRCA2*	BRCA2, DNA repair associated	Hs00609073_m1
*CCNA2*	Cyclin A2	Hs00996788_m1
*CCNH*	Cyclin H	Hs00236923_m1
*CDKN1A*	Cyclin dependent kinase inhibitor 1A	Hs00355782_m1
*CDKN2B*	Cyclin dependent kinase inhibitor 2B	Hs00793225_m1
*EFEMP1*	EGF containing fibulin like extracellular matrix protein 1	Hs00244575_m1
*EPHA4*	EPH receptor A4	Hs00953178_m1
*ERBB3*	Erb-b2 receptor tyrosine kinase 3	Hs00176538_m1
*GADD45B*	Growth arrest and DNA damage inducible beta	Hs00169587_m1
*GAS5*	Growth arrest specific 5 (non-protein coding)	Hs05021116_g1
*HDAC6*	Histone deacetylase 6	Hs00997427_m1
*HIST1H4B*	Histone cluster 1, H4b	Hs00374342_s1
*HIST2H4A; HIST2HB*	Histone cluster 2, H4a	Hs00269118_s1
*HUS1*	HUS1 checkpoint clamp component	Hs00189595_m1
*ITGB8*	Integrin subunit beta 8	Hs00174456_m1
*RPL13A*	Ribosomal protein L13a	Hs04194366_g1
*RPSA*	Ribosomal protein SA	Hs03046712_g1
*TAF1D*	TATA-box binding protein associated factor, RNA polymerase I subunit D	Hs00225533_m1
*TBRG4*	Transforming growth factor beta regulator 4	Hs01056250_g1
*TGFB2*	Transforming growth factor beta 2	Hs00234244_m1
*HPRT1*	Hypoxanthine phosphoribosyltransferase 1	Hs02800695_m1
*NAIP*	NLR family apoptosis inhibitory protein	Hs03037952_m1
*CASP4*	Caspase 4	Hs01031951_m1
*IKBKE*	inhibitor of kappa light polypeptide gene enhancer in B-cells, kinase epsilon	Hs01063858_m1
*PPARA*	peroxisome proliferator activated receptor alpha	Hs00947536_m1
*TP53*	tumor protein p53	Hs01034249_m1
*KRT1*	keratin 1	Hs00196158_m1
*KRT10*	keratin 10	Hs00166289_m1
*KPNA2*	karyopherin subunit alpha 2	Hs00818252_g1
*GAPDH*	Glyceraldehyde-3-phosphate dehydrogenase	Hs99999905_m1

The samples were prepared for PCR by mixing 55 μL of cDNA with 55 μL of TaqMan™ Universal PCR Master Mix, no AmpErase™ UNG (Applied Biosystems, Germany). Then, each fill reservoir of a TaqMan Array Card (Applied Biosystems, Germany) were loaded with 100 μL of sample-specific PCR reaction mix. Three wells were allocated per gene in this experiment. The array cards were run in the QuantStudioTM 12K Flex Real-time PCR system (Applied Biosystems, Germany) under the following conditions: 50°C for 2 min, 95°C for 10 min, then 40 cycles of 95°C for 15s and 60°C for 60s. All samples were run in triplicates.

### 2.9. Real-time quantitative polymerase chain reaction (RT-qPCR)

Comparative relative quantification was performed on prepared cDNA using the StepOnePlusTM Real-Time polymerase chain reaction (PCR) system (Applied Biosystems) and Taqman Gene Expression assays following protocols from the manufacturer (Applied Biosystems) for 4 genes (*ΔNp63α*, *TJP1*, *CLND1* and *OCLN*) (Table 2). All the samples were run in triplicates (each reaction: 2.0 μL cDNA, total volume 20 μL). The thermo cycling parameters were 95°C for 10 min followed by 40 cycles of 95°C for 15 s and 60°C for 1 min.

**Table 2:** The list of primers used in gene expression analyses using RT-qPCR.

Gene Symbol	Gene Name	TaqMan Assay ID
*ΔNp63α*	Tumor protein p63	Hs00978343_m1
*TJP1*	Tight junction protein 1	HS01551861_m1
*CLND1*	Claudin1	HS00221623_m1
*OCLN*	Occludin	Hs00170162_m1
*ERBB3*	Erb-b2 receptor tyrosine kinase 3	Hs00176538_m1

### 2.10. Statistical analysis

The analysis of gene expression data was performed using the Relative Expression Software Tool (REST^©^, Relative Expression Software Tool, Weihenstephan, Germany). REST implements the Pair Wise Fixed Reallocation Randomization Test^©^ to investigate the significance of changes in gene expression [34]. The Mann-Whitney U test was performed using GraphPad Prism 6.0 (GraphPad Software, San Diego, CA) in the analysis of results from the cell viability experiment [35, 36]. A *p* value of ≤ 0.05 was considered to be significant. The data are presented as the mean ± standard error.

## 3. Results

### 3.1. Examination of cell morphology

**Fig 1:**
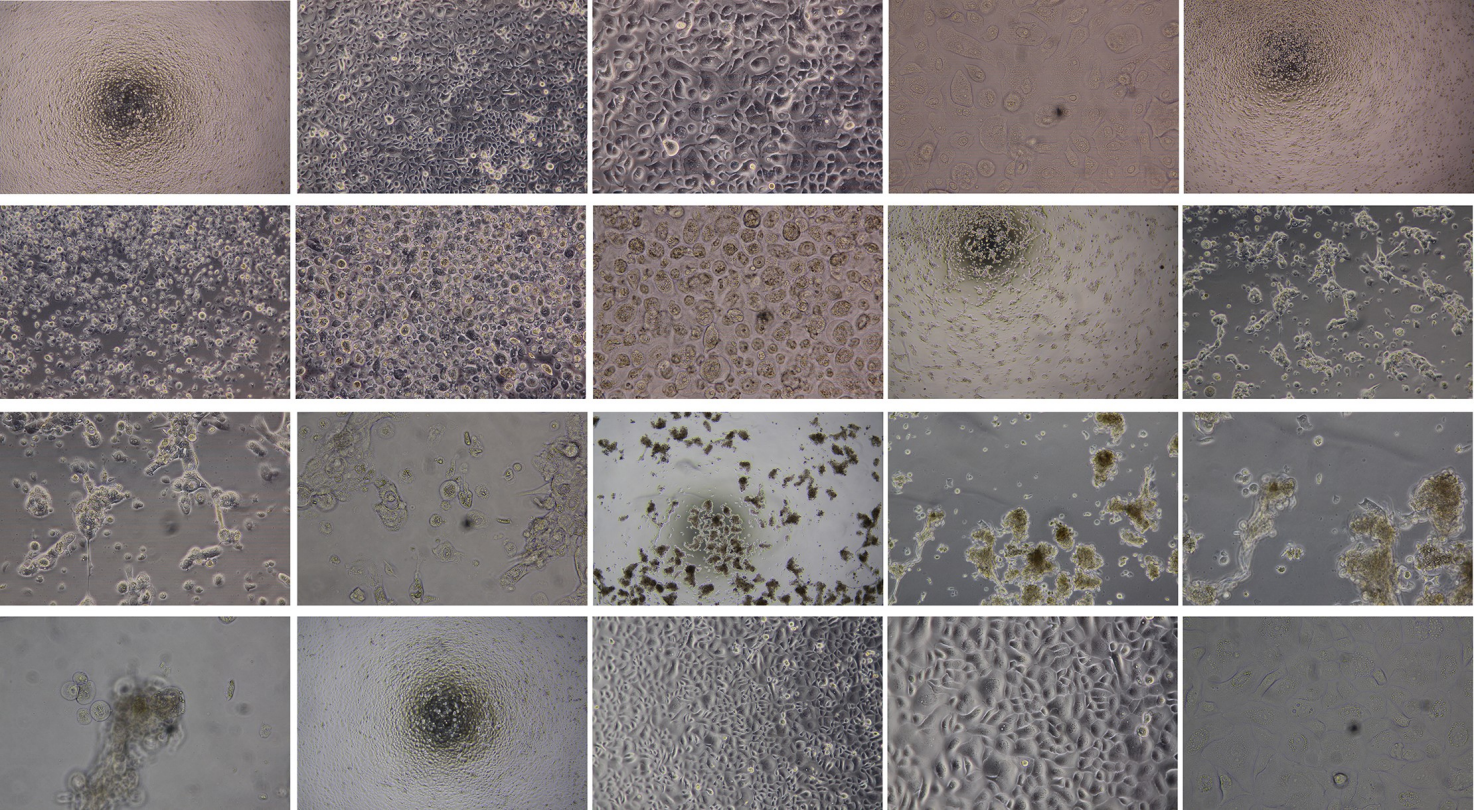
Light microscope images (4×, 10×, 20× and 40× magnification) of human oral mucosal epithelial cells incubated for 2 weeks at four storage temperatures (4°C, 12°C, 24°C and 37°C) and non-stored control. Morphology of cells at 4°C was most similar to control, although cell separation in some spots resulted in deviation from uniform polygonal morphology. Cells mainly held rounded morphology at 12°C, whereas the typical cobblestone appearance at confluence was totally disappeared at 24°C. Most of cells were detached at 37°C and formed cell clusters.

The confluent monolayer culture of OMECs presented typical cobblestone appearance under light microscope before storage (Fig 1). A two-week cultured OMECs at 4°C resulted in morphological features resembling non-stored baseline control cells. However, uneven cell-cell contact in some spots led to cell separation and deviation from uniform polygonal morphology. Two weeks after the established incubation period, phase contrast micrographs of cells stored at 12°C has identified mainly cells with rounded morphology. At 24°C, the mosaic-like growth pattern typical of epithelial cells at confluence had completely disappeared. Most of the cells were detached at 37°C and formed cell clusters. The detached and/or loosened cells at 4°C, 12°C, 24°C and especially 37°C were easily washed away during medium change.

**Fig 2:**
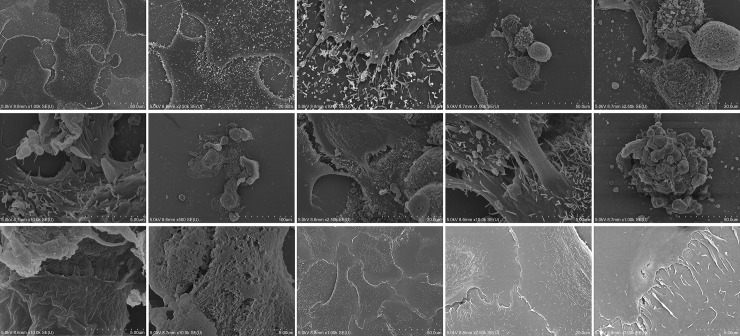
Scanning electron micrographs of human oral mucosal epithelial cells incubated for 2 weeks at four storage temperatures (4°C, 12°C, 24°C and 37°C) and non-stored control. The cells in control were closely attached to each other with distinct cell borders (Arrow). Cells at 4°C retained the most similar morphology compared to the control, but there was evidence of cell separation between some adjacent cells (Square). Clustered cells formed uneven islands at 12°C and 24°C and blebbing morphology was seen on some of the cells (Arrow). At 37°C, the remaining irregular and shrunken cells were surrounded by debris.

The evaluation of cell-cell contacts and cell adhesion of non-stored baseline OMECs using SEM demonstrated a uniform, closely attached and solid monolayer of cells with distinct cell borders (Fig 2). At 4°C, epithelial cells retained extended filopodia, enlarged flattened appearance and microvilli on the apical surface similar to non-stored baseline control cells, but there was evidence of cell separation between some adjacent cells. The clustered cells, forming uneven islands, at 12°C and 24°C resulted in apparent changes in cultured epithelial cells morphology. Blebbing was also seen on some of the cells. At 37°C, the remaining irregular, shrunken and disintegrated cells were surrounded by debris.

### 3.2. Assessment of cell viability

**Fig 3:**
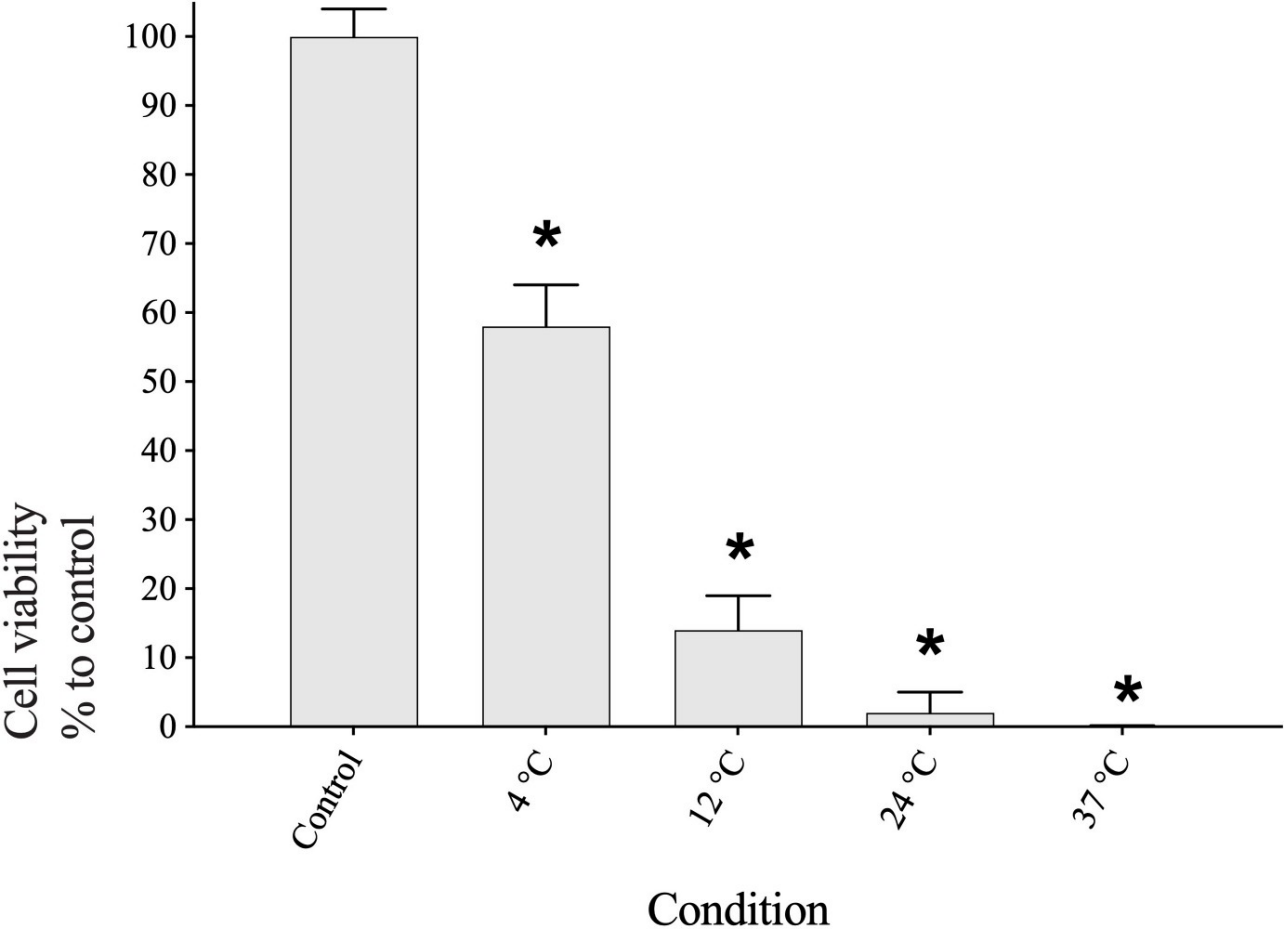
Cell viability of human oral mucosal epithelial cells sheets incubated for 2 weeks at four storage temperatures (4°C, 12°C, 24°C and 37°C) relative to non-stored control. Asterisk (*) above the bar denotes significant differences (*p* ≤ 0.05) as compared with the non-stored control.

The viability of OMECs incubated for two weeks was decreased with increasing storage temperature compared to non-stored baseline control cells (Fig 3). The 4°C experimental group with 58% viability had the highest number of living cells, whereas the 37°C group recorded no living cells. The 12°C and 24°C study groups, presented 14% and 2% viability, respectively. The cell viability in all groups was significantly lower than baseline control (*p* ≤ 0.05).

### 3.3. Evaluation of gene expression

**Fig 4:**
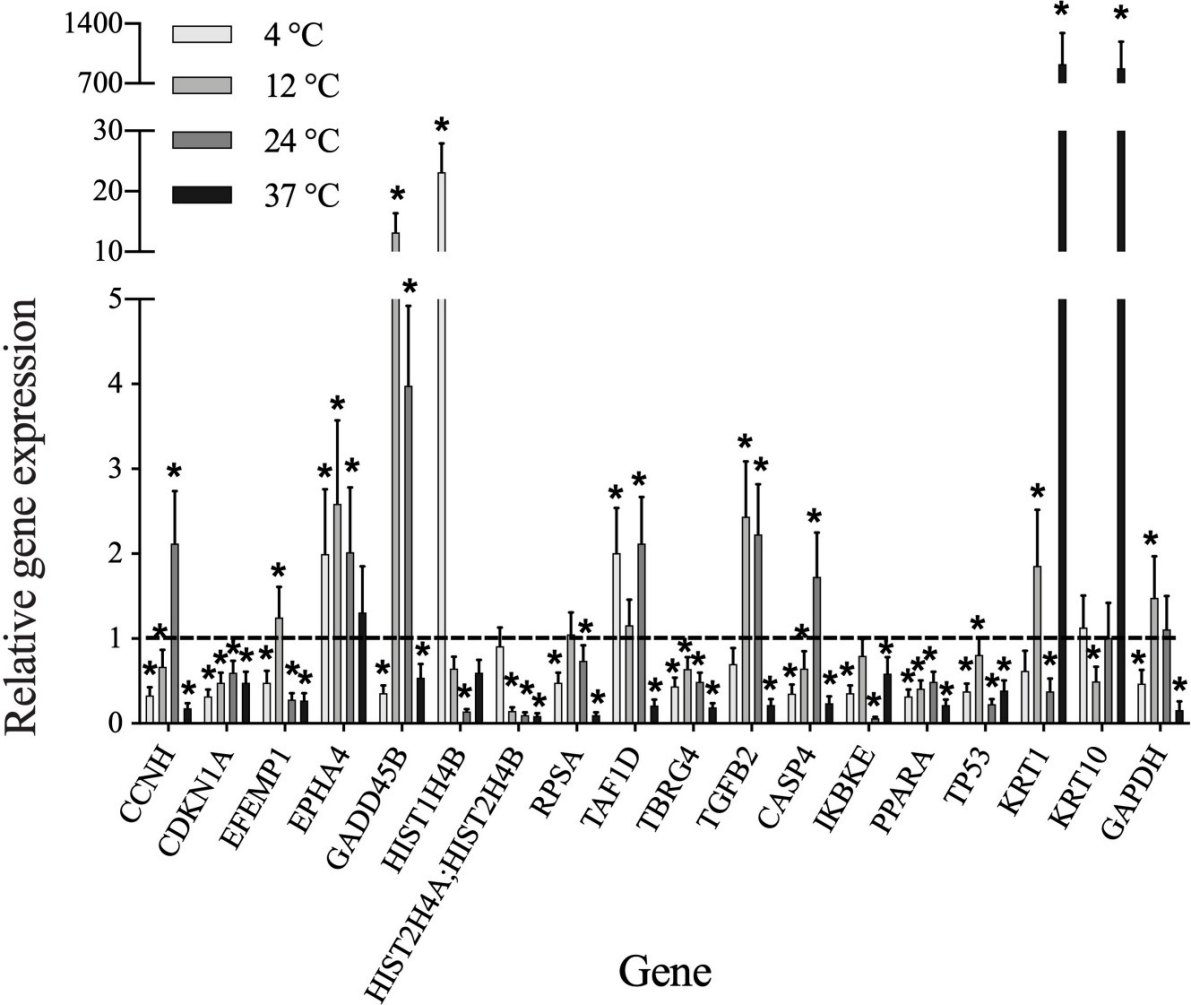
TaqMan array cards analysis of the expression of 18 genes in human oral mucosal epithelial cell sheets incubated for 2 weeks at four storage temperatures (4°C, 12°C, 24°C and 37°C) relative to non-stored control. Asterisks (*) above the bar denote significant differences (*p* ≤ 0.05).

This study considered a log2-fold change significant at thresholds above 1 and below -1 (i.e., > two-fold change). Moreover, this approach was only applied to 4°C, 12°C and 24°C groups as all cells died upon storage at 37°C (Supplementary). Thus, altered expression at this temperature was deemed irrelevant. We identified 18 differentially expressed genes upon storage using the TaqMan Gene Expression Array (Fig 4). Four of them (*CDKN1A*, *TBRG4*, *PPARA* and *TP53*) were downregulated in all four different temperature conditions when compared to non-stored baseline control (*p* ≤ 0.05). Four other genes were also downregulated at 4°C and 37°C, with upregulation at 12°C (*EFEMP1*), 24°C (*CCNH* and *CASP4*), or both 12°C and 24°C (*GADD45B*). Similarly, significant downregulation for all 4 temperature conditions, excluding insignificant changes at 4°C (*HIST2H4A; HIST2HB*) and 12°C (*RPSA* and *IKBKE*), were also observed. In contrast, *EPHA4* showed significant upregulation at 4°C, 12°C and 24°C. Six genes (*HIST1H4B*, *TAF1D*, *TGFB2*, *KRT1*, *KRT10* and *GAPDH*) presented a combination of significant (both downregulation and upregulation) as well as insignificant changes in expression in studied groups of each different temperature conditions.

**Fig 5:**
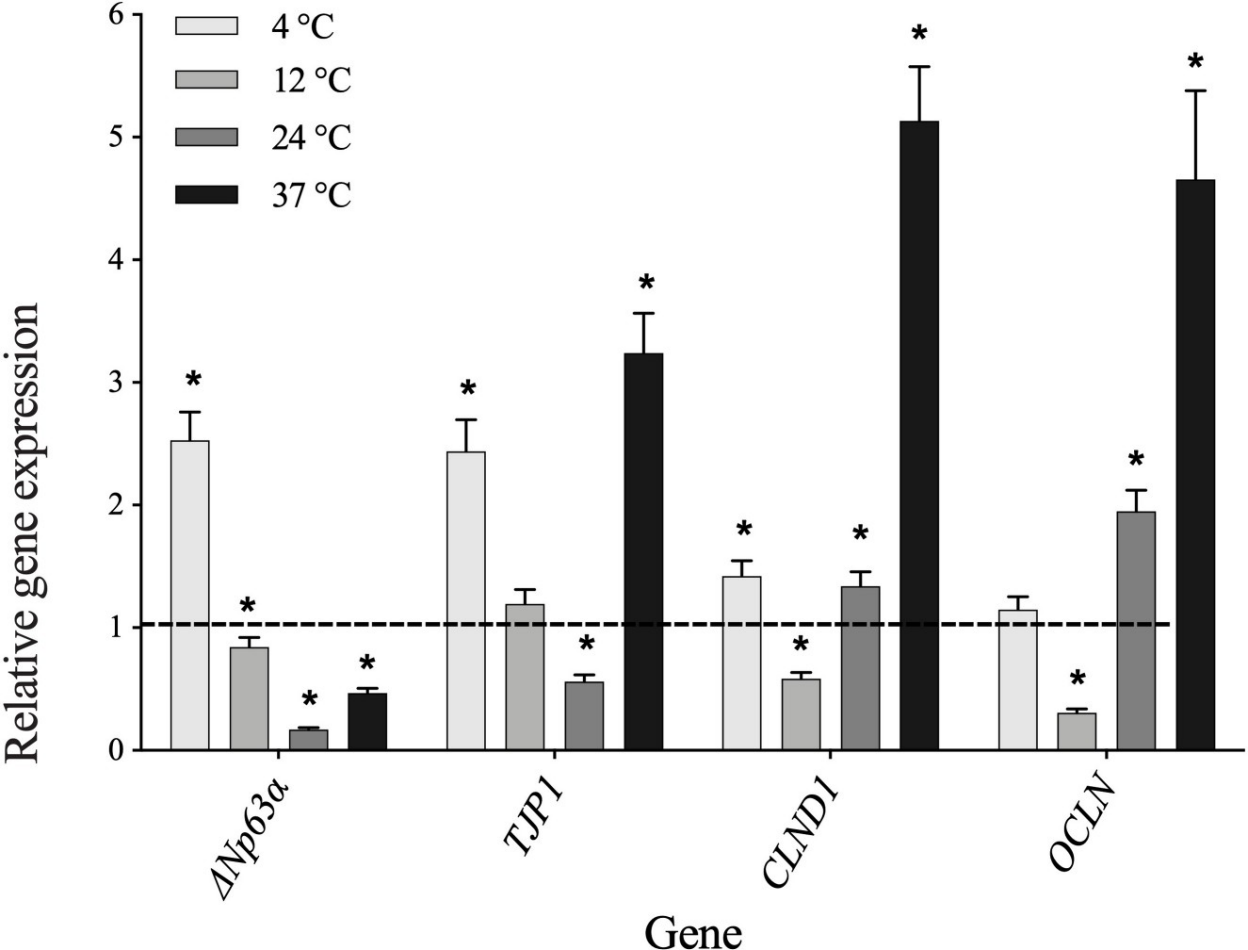
RT-qPCR analysis of the expression of 4 genes in human oral mucosal epithelial cell sheets incubated for 2 weeks at four storage temperatures (4°C, 12°C, 24°C and 37°C) relative to non-stored control. Asterisks (*) above the bar denote significant differences (*p* ≤ 0.05).

Results from RT-qPCR (Fig 5) indicated that *ΔNp63α*, *TJP1* and *CLND1* were significantly upregulated at 4°C but *OCLN* remained unchanged. At 12°C, three of genes (*ΔNp63α*, *CLND1* and *OCLN*) were significantly downregulated, except unchanged *TJP1*. At 24°C, significant downregulation was observed for *ΔNp63α* and *TJP1*, whereas upregulation for *CLND1* and *OCLN*.

## 4. Discussion

To establish optimal storage conditions for OMECs to maximize transplantation success and extend access to regenerative medicine, this study aimed to assess the morphology, cell viability and expression of selected genes of cultured OMECs stored at four different temperatures (4°C, 12°C, 24°C and 37°C) for two weeks. The overall morphology and ultrastructure of cells stored at 4°C was most similar to non-stored baseline controls, whereas the 37°C group was the most dissimilar ones with only dead cells. The presence of cell separation between some adjacent cells at 4°C group and prominent morphological deformation, shrinkage, and membrane blebbing at higher temperatures in our study were also observed by others, Islam et al [16] and Jackson et al [15] for cultured oral keratinocytes and epidermal cells after one and two weeks of storage, respectively. However, the authors found that mid range temperature groups of 12°C, 16°C and 20°C resulted in the best preserved morphology.

Cells stored at 4°C had the highest viability rate (58%) compared to other three experimental groups with higher temperatures, whereas the 37°C group presented no living cells. The former is in the viability range of suspended human oral keratinocyte (~63%) in DMEM, assessed using trypan blue exclusion test, after three days storage at 4°C [37]. Comparably, Islam et al [16] showed that adherent human oral keratinocytes cultured in HEPES-MEM had ~45% viability, as measured by calcein-acetoxymethyl ester fluorescence, following one week storage at 4°C. The highest viability of the latter study, however, belonged to 12°C group (~85%).

Other cell types have also been studied for the effect of storage temperature. For example, human retinal pigment epithelium [38], epidermal keratinocytes [15] and conjunctival epithelium [39] recorded ~4% (one week), ~18% (two weeks) and ~72% (one week) viable cells at 4°C, respectively. For those cell types, the viability was greatest at 16°C (~48%,), 24°C (~97%,) and 12°C (~95%), respectively. This variation may be due to the cell types, the culture medium, storage period or the viability assay used. Nevertheless, the survival of donor cells on the ocular surface after limbal stem cells allografts has been found not to correlate with clinical outcomes [40]. Additionally, hypothermia (2–8°C) is a rather commonly used storage temperature prior to transplantation [41].

Our gene analysis revealed that *GAPDH*, an important enzyme for energy metabolism, was affected by temperature. Significant *GAPDH* downregulation was only observed at 4°C (excluding 37°C with no viability). Similar response has also been demonstrated for corneal cultures [42] and oral keratinocytes [16]. Based on Arrhenius equation, the principle behind hypothermic storage is that the low temperature reduces chemical reaction rates and hence declines cellular metabolic activities, which lowers the demand for oxygen and substrates [43]. The temperature dependant metabolic status, indicated as lactate/glucose ratio, has been previously shown to be inversely correlated with oxygen tension in epidermal keratinocytes [15]. Additionally, the glycolytic pathway has been suggested to play a major role in keratinocyte energy production, regardless of available oxygen level [15, 44–46]. For human oral keratinocytes, Islam et al [16] suggested glycolysis could be responsible for at least partial energy production at 4–37°C after one week of storage. *GAPDH* is widely used as a housekeeping gene for RT-qPCR or loading control for western blot. Therefore, researchers should be mindful in selecting appropriate endogenous controls in molecular research associated with temperature. In the present study, *ERBB3* was used as reference gene because its expression level was unaffected by experimental factors.

Histones are positively charged basic nuclear proteins that play important structural and functional roles in the transition between active and inactive chromatin states through compacting DNA [47]. Hence, they play a central role in transcription regulation, DNA repair, DNA replication and chromosomal stability [48]. In this study, *HIST1H4B*, one of the histone cluster genes, was highly upregulated at 4°C. *HIST2H4A/B*, another member of the same family, was not significantly different at 4°C when compared to non-stored baseline control. In accordance with our finding, Raeder et al. [49], have reported the upregulation of *HIST1H4B* in culture of human limbal epithelial cells stored at 4°C for 2–7 days. Low temperature may induce expression of this histone H4 encoding gene in order to acclimatize the cells to hypothermia by stabilizing nucleosomes and repressing transcription, even after 2 weeks storage.

The effect of temperature on stored OMECs was also observed in the expression of genes involved in cell adhesion, migration, motility and differentiation. *EFEMP1* was upregulated at 12°C. As a member of the fibulin family of extracellular glycoproteins, it is associated with multiple structural and functional roles including morphology, growth, adhesion and motility [50]. *RPSA*, another multifunctional gene (involved in e.g., cell adhesion, differentiation and migration), remained unchanged at 12°C but was downregulated at all other temperature conditions. Finally, *EPHA4* was significantly upregulated at 4°C, 12°C and 24°C. Its key functions are the maintenance of the basement membrane and the integrity of the extracellular matrix [51]. Among genes used as differentiation markers, *PPARA* and *TP53* were downregulated at all four temperature conditions. *CDKN1A*, alias p21, responded in the same fashion. This gene has been previously shown to be under the transcriptional control of p53 [52]. Two other differentiation markers, *KRT1* and *KRT10*, were either unchanged (4°C) or downregulated (24°C and 12°C, respectively), except an upregulation at 12°C for *KRT1*. The 37°C group was not evaluated due to a majority of dead cells. The expression pattern of differentiation markers following storage have been previously reported by our group in other cultured epithelial cells [14, 38, 53]. Using immunostaining, Islam et al [16] presented similar results for putative stem cell markers in oral keratinocytes stored for one week at 4°C, 12°C and 20°C. These results indicated that the expression levels of some adhesion-, growth- and apoptosis-related genes were favourable for cultured epithelial cells at 4°C–24°C.

The expression level of *ΔNp63α*, a stemness marker for OMECs [54, 55], was significantly upregulated at 4°C compared to other experimental groups. The clinical success in treatment of LSCD has been linked to the high number of cells with *ΔNp63α* expression in transplantation of cultured LSC sheets [20]. The satisfactory results have been observed by authors in 78% of patients when LSCs cultures expressed more than 3% *ΔNp63α*. Another important factor in transplantation is the maintenance of barrier function of cultured sheet [56], which is partly defined by cell membrane and cell junctions [57]. For example, Claudin and occludin, members of tight junctions transmembrane proteins, interact with zonula occludens proteins such as *TJP1* (alias *ZO-1*) on cell’s plasma membrane face for anchoring them to the actin cytoskeleton [58]. These markers have previously been examined in human and rabbit cultivated OMEC sheets [56]. Our study indicated that genes associated with tight junction were either upregulated (*TJP1* and *CLND1*) or unchanged (*OCLN*) at 4°C. Additionally, the culture of OMECs presented upregulation of *CLND1* and *OCLN* at 24°C. Although 37°C led to highest expression level of tight junction-associated genes, storage of OMECs for two weeks is disadvantageous due to the dominance of dead cells, downregulation of stemness gene (*ΔNp63α*) and overexpression of keratinization markers (*KRT1* and *KRT10*).

The upregulation of *GADD45B*, stress-induced growth arrest gene, is indicative of the high level of stress imposed on OMECs after two weeks of storage at 12°C and 24°C. *GADD45B* is considered a crucial factor in DNA repair, cell survival, growth arrest, apoptosis and DNA demethylation [59–61]. Similarly, *TGFB2* showed upregulated levels at the same storage temperatures, whereas *CCNH* was only upregulated at 12°C. *TGFB2* belongs to a group of proteins that regulate several cellular functions, especially inhibition of cell proliferation through cell cycle arrest in G1 phase [62, 63]. Its regulatory role is mediated by a series of formation, activation and subsequent inactivation of the G1 cyclin-dependent kinase (CDK) complexes, another group of proteins that are partly controlled by cyclins (e.g., *CCNH*) [64, 65]. The levels and activities of both G1 cyclins and CDKs are directly affected by *TGFB2* [66, 67].

One unanticipated finding was the upregulation of *TAF1D*, a histone-relevant transcriptional regulation gene, at 4°C and 24°C. It is in contrast to downregulation of *TBRG4*, also termed cell cycle progression restoration protein 2, in all four experimental groups. *TAF1D*, the largest subunit of *TFIID*, is an essential component of transcription machinery which acts through harboring bromodomains and histone acetyltransferase activity [68, 69]. It is difficult to explain this result, but it might be related to the association revealed between *TAF1D* and genotoxic/oxidative stress-induced apoptosis [70]. In this context, we observed upregulated stress-induced growth arrest gene (*GADD45B*) at 24°C. Moreover, the apoptosis-associated gene *CASP4* was also upregulated at 24°C, in contrast to other downregulated groups. This was supported by our photomicrographs illustrating cells with membrane blebbing, indicative of apoptosis [71]. However, others have reported cold storage-induced apoptosis and/or necrosis in oral keratinocytes [16] and epithelial cells [71].

The list of 22 regulated genes was subjected to DAVID Functional Annotation Tool (https://david.ncifcrf.gov/) to test for over-representation in functional pathways. Unsurprisingly, Cell Cycle came out as the most significantly affected in the KEGG PATHWAY Database, and the only relevant, sustaining significance after adjustment for multiple testing by the Benjamini–Hochberg method [72] (*p* = 3.4E-3, and the 5 genes: *CCNH*, *CDKN1A*, *GADD45B*, *TGFB2* and *TP53)*. If more experiments had been performed, the statistical power would increase, and we would probably find more affected genes as well as signaling pathways giving further insight in the cellular processes affected upon storage.

Our study had some limitations. We decided not to adjust *p*-values for multiple testing to avoid false negative findings (type II statistical errors), at the expense of increasing the likelihood of including false positives (type I statistical errors). Another limitation was the use of cells from a single donor, at the possible risk of systemic factors’ influence (e.g. donor health and age) on extrapolation of the results [73–75].

The results of morphology, cell viability and gene expression assays in the current study showed that 4°C is the most appropriate temperature to store OMECs for up two weeks. Similarly, Utheim et al [32] found that storage at 4°C and 12°C are more suitable for transplantation than 37°C after one-week culture of oral keratinocytes. However, these results differ from a previous study reporting 12°C and 16°C as optimal temperatures for cultured oral keratinocytes to maintain their structure and function after one week of storage [16]. Another similar study also found 12°C and 24°C superior for storage of cultured epidermal cells for two weeks [15]. Whilst our findings did not confirm their conclusions, it did support refrigeration (2°C–8°C), one of the current methods employed in the storage of cultured epithelial cells [41]. Although this simple technique may be practical for short-term storage, several drawbacks such as limited shelf-life, high cost, risk of contamination and genetic drift have discouraged its application for long-term preservation [76].

## 5. Conclusions

This study found that the morphology of cultured human OMECs stored at 4°C for two weeks was most similar to non-stored baseline controls. Additionally, cells stored at 4°C had the highest viability rate (58%) when compared to other temperatures experimental groups. Gene analysis also revealed some levels of interruption at 12°C and/or 24°C through e.g., stress-induced growth arrest (*GADD45B*) and cell proliferation inhibition genes (*TGFB2*). However, some genes involved in cell adhesion, migration, motility and differentiation maintained transcription levels beneficial for cultured epithelial cells at 4°C–24°C. Taken together, these results suggest that storing OMECs at 4°C for two-week is the most appropriate temperature with the highest viability rate and morphology rather similar to pre-stored OME cells. Therefore, it is most likely to be the best storage conditions for clinical transplantation.

## Supporting information

S1 Table The list of 30 genes examined using the TaqMan gene expression array in this study. Among 30 studied genes, 12 were omitted (non-bolded genes). This study considered a log2-fold change significant at thresholds over 1 and under -1 (i.e., > two-fold change). Additionally, this approach was only applied to 4°C, 12°C and 24°C groups as all cells died upon storage at 37°C.
(XLSX)

S2 Table The list of 4 genes examined using RT-qPCR in this study. Among 30 studied genes, 12 were omitted (non-bolded genes). This study considered a log2-fold change significant at thresholds over 1 and under -1 (i.e., > two-fold change). Additionally, this approach was only applied to 4°C, 12°C and 24°C groups as all cells died upon storage at 37°C.
(XLSX)
